# Maternal Inflammation, Fetal Brain Implications and Suggested Neuroprotection: A Summary of 10 Years of Research in Animal Models

**DOI:** 10.5041/RMMJ.10305

**Published:** 2017-04-28

**Authors:** Yuval Ginsberg, Nizar Khatib, Zeev Weiner, Ron Beloosesky

**Affiliations:** 1 Department of Obstetrics and Gynecology, Rambam Health Care Campus, Haifa, Israel; 2 The Ruth & Bruce Rappaport Faculty of Medicine, Technion–Israel Institute of Technology, Haifa, Israel

**Keywords:** Fetal brain injury, magnesium sulfate, maternal inflammation, N-acetyl cysteine neuroprotection

## Abstract

A growing body of evidence implies that maternal inflammation during pregnancy is associated with increased risk of neurodevelopmental disorders in the offspring. The pathophysiological mechanisms by which maternal inflammation evokes fetal brain injury and contributes to long-term adverse neurological outcomes are not completely understood. In this review, we summarize 10 years of our research experience on maternal inflammation and the implications upon the fetal/offspring brain. We review our findings regarding the underlying mechanisms that connects maternal inflammation and fetal brain injuries (e.g. cytokines, oxidative stress); we discuss our imaging, pathological and behavioral test results which support brain damage following maternal inflammation; and finally we describe some of the therapeutic strategies which might prevent the damage.

## INTRODUCTION

For many years, intrapartum hypoxia/asphyxia was considered the main etiology for cerebral palsy and fetal brain injuries. In reality, new epidemiological studies have revealed that asphyxia accounts for only 20% of the cases of cerebral palsy, and 70% of the cases arise even before the onset of labor.^1^ In recent years, a plethora of both clinical and epidemiological studies has established a correlation between an “adverse” intrauterine environment and abnormal development of the fetal brain. While the consequences of chorioamnionitis for both cerebral palsy and adverse neurologic injuries are clear,^2^–^6^ maternal inflammation that occurs during critical periods of fetal development has also been identified as a significant risk factor for some neuropsychiatric and neurobehavioral disorders, including schizophrenia, autism and cognitive delay.^7^–^13^ However, the mechanism by which maternal infection and/or inflammation of the uterine cavity can evoke fetal brain injury and contribute to long-term adverse neurological outcomes remains unclear. This is a summary of our 10-year^14^–^23^ research using a rat model of maternal inflammation for a better understanding of the mechanisms by which maternal inflammation/infection may attenuate normal fetal brain development, and optional neuroprotective interventions.

## CHOOSING THE RIGHT MODEL

### Systemic Versus Local Inflammation

To understand the mechanisms associated with human fetal brain injury, a model with similar developmental milestones and brain structure proportions should be found. The insult should be delivered at an equivalent stage of intrauterine brain development for an identical period of vulnerability. Measurement of the outcomes of the insult should be feasible by acceptable methods. Clearly, no animal model can fully mimic the development of the human brain, and each species has its own characteristics.

Several types of animal models have been suggested for elucidating the mechanisms that link prenatal inflammation and adverse fetal brain development.^20^,^24^–^28^ Each animal model has its own clinical advantages. Rodent models have been used widely to investigate the influence of local insult on preterm delivery and fetal brain development.^25^,^27^–^31^ Most of these studies used local delivery of an insult (uterine injection of lipopolysaccharide [LPS], for example) to mimic a well-documented mechanism of ascending intrauterine infection, which might cause preterm birth or chorioamnionitis. Systemic models that mimic clinical scenarios of maternal infection/inflammation during gestation have received less attention.

### Timing of the Insult

The development and maturation of the human brain is a complex and continuous process. Primary neurulation occurs during weeks 3–4, neuronal proliferation during months 3–4, migration during months 3–5, and myelination begins during the second trimester and continues into adulthood. Hence, as mentioned above, the timing of an insult is crucial to compare and evaluate the neurodevelopmental response of offspring. While early insult is associated with structural brain abnormalities such as neural tube defects (primary neurulation), later insults may disrupt the migration process of post-mitotic neurons and lead to aberrant cortical development.^28^ Late-gestation insults have been found to associate more with cognitive, behavioral, and psychiatric disorders, such as schizophrenia, autism, and obsessive compulsive disorders.^27^,^32^,^33^

## MECHANISM OF INJURY

Both epidemiological and experimental studies in animals have demonstrated that maternal infection can damage the developing brain.^34^–^37^ It was suggested that infections activate inflammatory pathways, causing the release of various proinflammatory biomarkers, such as cytokines, interleukins, and other molecules. Cytokines are intracellular mediators that are crucial to counter infections. While proinflammatory cytokines mobilize immune system cells to proliferate, to produce more cytokines, and to encourage the inflammatory cascade, anti-inflammatory cytokines act to depress and control the inflammatory response. Since cytokines are 50 kDa, the potential transfer of cytokines between a mother, a fetus, and amniotic fluid might occur.

**Figure 1:**
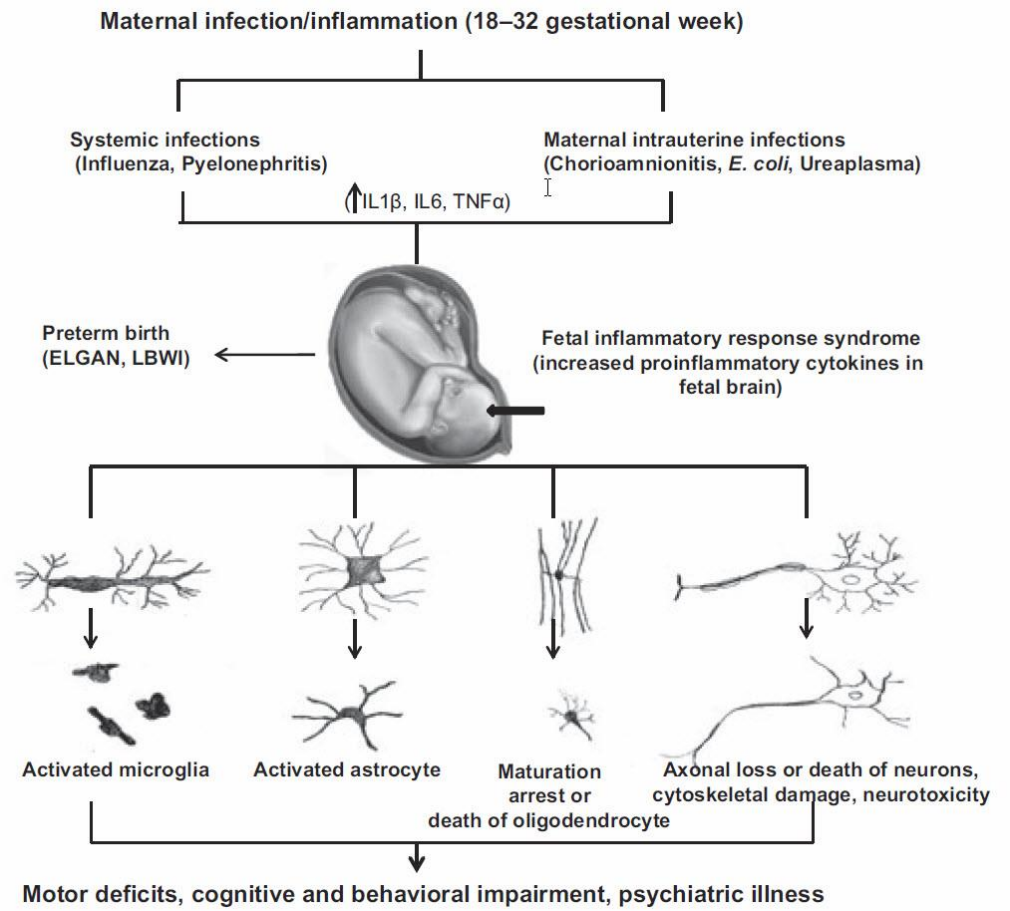
Suggested Mechanism of Fetal Brain Injury Following Maternal Inflammation. Probable mechanisms of fetal brain injury with *in utero* exposure to maternal inflammation. Reprinted from Burd et al.,^28^ Copyright (2012), with permission from John Wiley and Sons, all rights reserved. ELGAN, extremely low gestation neonates; LBWI, low birth weight infants.

Proinflammatory cytokines such as tumor necrosis factor-alpha (TNF-α), interleukin (IL) 1β, and IL-6 from astrocytes and microglia may directly damage oligodendrocytes and neurons. For example, the injection of IL-1β leads to neuronal death and delayed myelination in neonatal rats.^38^ Tumor necrosis factor-α induces cell death in mature oligodendrocytes and apoptosis in developing oligodendrocytes.^39^,^40^ Exposure to TNF-α appears to be associated with reduced myelination and diffuse white matter damage in fetal rodents^24^ and in preterm infants.^41^ The production of free radicals following the initial neural injury exacerbates collateral damage for neurogenesis and neurodifferentiation (Figure 1).^28^,^37^,^42^

## MEASURING THE CYTOKINE RESPONSE

In light of the potential contribution of cytokines to fetal neurological injury, we first sought to evaluate fetal inflammatory responses to maternal inflammation. One common method of mimicking inflammation associated with infection in animal models is through the exposure of modified proteins. Lipopolysaccharide is a Gram-negative bacterial component that mimics bacterial infections, and LPS exposure was found to induce the production of IL-6, IL-1β, and TNF-α. We used intraperitoneal (i.p.) injections of LPS to induce maternal systemic inflammation in pregnant rats.^16^–^18^,^21^,^28^,^43^

**Figure 2:**
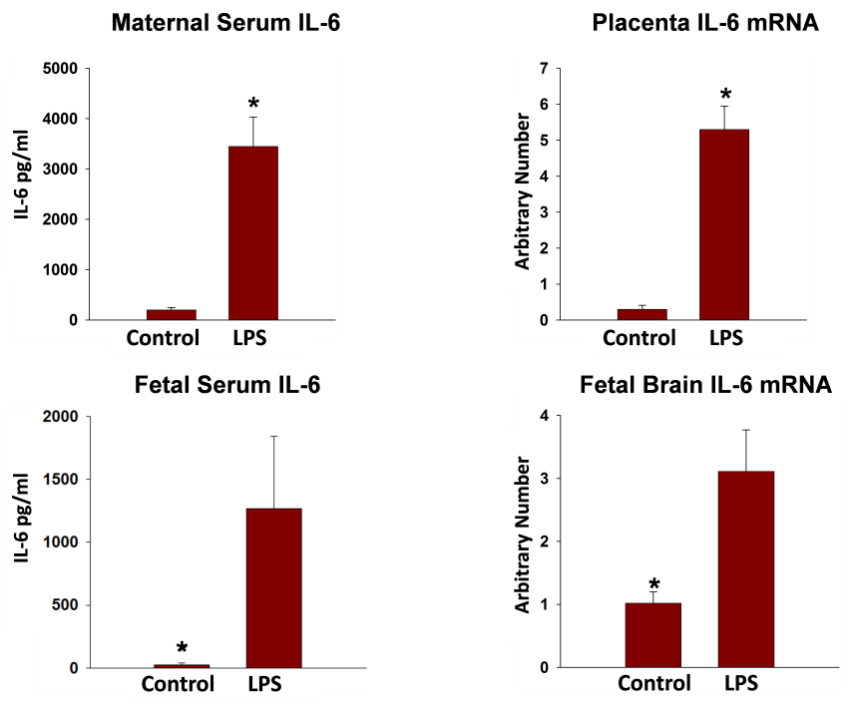
Maternal Serum, Placenta, Fetal Serum, and Fetal Brain Levels of IL-6 Following Maternal Inflammation. Interleukin-6 levels in the maternal serum, placenta, fetal serum, and fetal brain, 3–4 hours following maternal inflammation induced by LPS. *P<0.05, significant difference between control and LPS-induced maternal inflammation.

In response to maternal i.p. injections of LPS, TNF-α was the first cytokine to peak; this was followed by a quick return to baseline in both maternal circulation and amniotic fluid. Interleukin-1β and IL-6 had delayed responses. Assessment of changes in the mRNA levels of these proinflammatory cytokines revealed that LPS induced increases in TNF-α, IL-6, and IL-10, both in the chorio-amnion and in the placenta. Similar results were demonstrated in the fetal brain (Figure 2).

Our results imply that acute maternal inflammation can affect the proinflammatory status of the amniotic fluid, and the chorio-amnion membranes, fetal serum, and brain. All of them are associated with fetal brain injury. Furthermore, we recognize that time does matter. In early gestation, the fetal anti-inflammatory response is decreased due to the immature immune system. Therefore, the earlier maternal inflammation occurs, the worse the prognosis.

## MEASURING THE OXIDATIVE STRESS RESPONSE

Increased maternal oxidative stress in pregnancy is associated with an increased risk of poor pregnancy outcome. Several studies have shown that markers of oxidative stress are increased during maternal inflammation. Following maternal LPS injection, elevated oxidative stress has been observed in rat fetus brains several hours and days after delivery.^14^,^22^,^44^,^45^ As previously mentioned, proinflammatory cytokines induce oxidative stress mediators. In a “vicious cycle” mechanism, oxidative stress rises, increasing cytokine induction. Using our rat model,^14^,^22^ we demonstrated that maternal inflammation induces oxidative stress in the maternal serum and amniotic fluid, and increases the basal oxidative state in neonates.

In another study,^15^ we demonstrated that LPS-induced maternal inflammation at 16 days of gestational (E16) and 18 days of gestation (E18) significantly increased fetal brain phosphor-neuronal nitric oxide synthase, nuclear factor kappa-light-chain-enhancer of activated B cells (NF-κB) p65, and chemokine (C-C motif) ligand 2 protein levels.

## FETAL BRAIN IMAGING

Our group pioneered the use of magnetic resonance imaging (MRI) to demonstrate the long-term consequences of maternal inflammation on fetal brain development. By using advanced MRI methods,^17^ such as T2 relaxation time, diffusion tensor imaging (DTI), and apparent diffusion coefficient (ADC), we demonstrated that offspring of LPS-treated dams, 25 days postnatal, exhibited significant changes in both white and gray matter (e.g. hypothalamus, motor cortex, corpus callosum, thalamus, hippocampus), consistent with diffuse cerebral injury.

## CLINICAL ASPECTS OF BRAIN INJURY—LEARNING AND MEMORY ABILITIES

**Figure 3:**
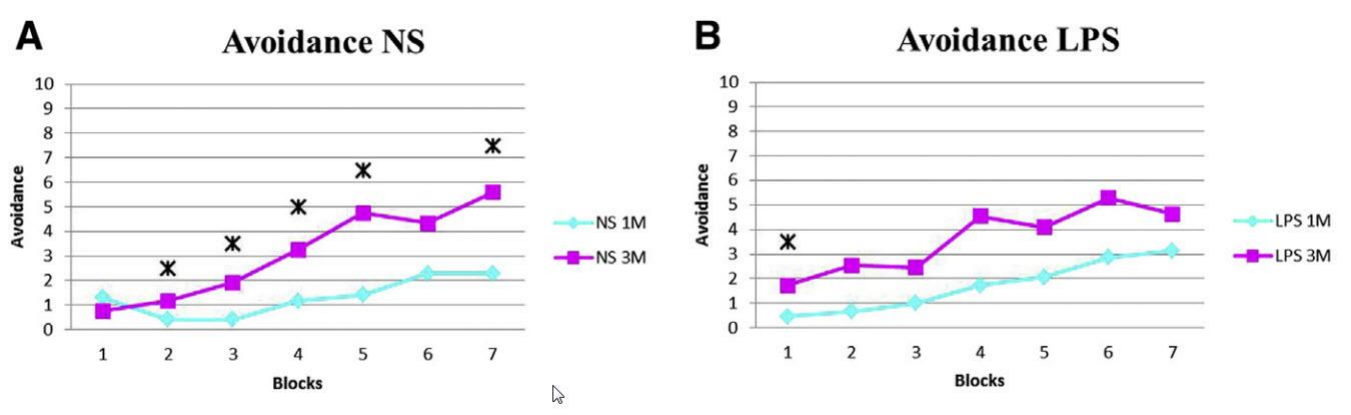
Neonate Learning Deterioration Following Maternal Inflammation. **(A)** Learning abilities of the NS group improved significantly between the first and the third months in almost every block (each block composed of 10 tests). **(B)** The LPS group exhibited no significant improvement in learning abilities with time. LPS, lipopolysaccharide; NS, normal saline; 1M, 1 month of age; 3M, 3 months of age. Reprinted from Lamhot et al.,^23^ Copyright (2015), with permission from Elsevier. Asterisks (*): Significance (*P*<0.05) in the learning abilities, examined by the avoidance test in each block between 1 and 3 months. **Blocks (X Axis):** Each test comprised 7 blocks of 10 stimulus cycles each. Tests were administered to each rat at 1 and 3 months. **Avoidance (Y Axis):** Number of positive avoidance responses in each block.

As evident by MRI, offspring of LPS-treated dams exhibited white and gray matter injury. Many of these areas were in regions known to be involved in learning and memory, including the dorsal striatum, medial septal nucleus, entorhinal cortex, and molecular dentate gyrus.^17^,^18^ To translate laboratory and imaging data into clinical practice, we decided to examine learning and memory abilities of neonates following maternal inflammation. We used the two-way shuttle avoidance box test to evaluate learning and memory abilities. Our data demonstrated that maternal inflammation significantly attenuates learning abilities of 3-month-old offspring (Figure 3).^23^

## THERAPEUTIC STRATEGIES OF NEUROPROTECTION

Since maternal infection/inflammation increases offspring brain cytokines and free radicals, both anti-inflammatory substances and free radical scavengers might have therapeutic properties that prevent fetal adverse neurologic outcomes.

### Magnesium Sulfate

During the last decade, several prospective studies in pregnant patients have demonstrated the neuroprotective effect of magnesium sulfate (MG) in preventing preterm white matter brain injury.^46^–^50^ Following the Cochrane meta-analysis review,^51^ MG became “the drug of choice” for preventing brain injuries and cerebral palsy in “at-risk” fetuses. In light of the importance of preventing newborn brain injury, and although the neuroprotective mechanism of MG has not been elucidated, we sought to investigate the use of MG in preventing short- and long-term fetal brain consequences of LPS-induced maternal inflammation.

#### Oxidative Stress and Inflammatory Modulators Following Magnesium Sulfate Administration

**Figure 4:**
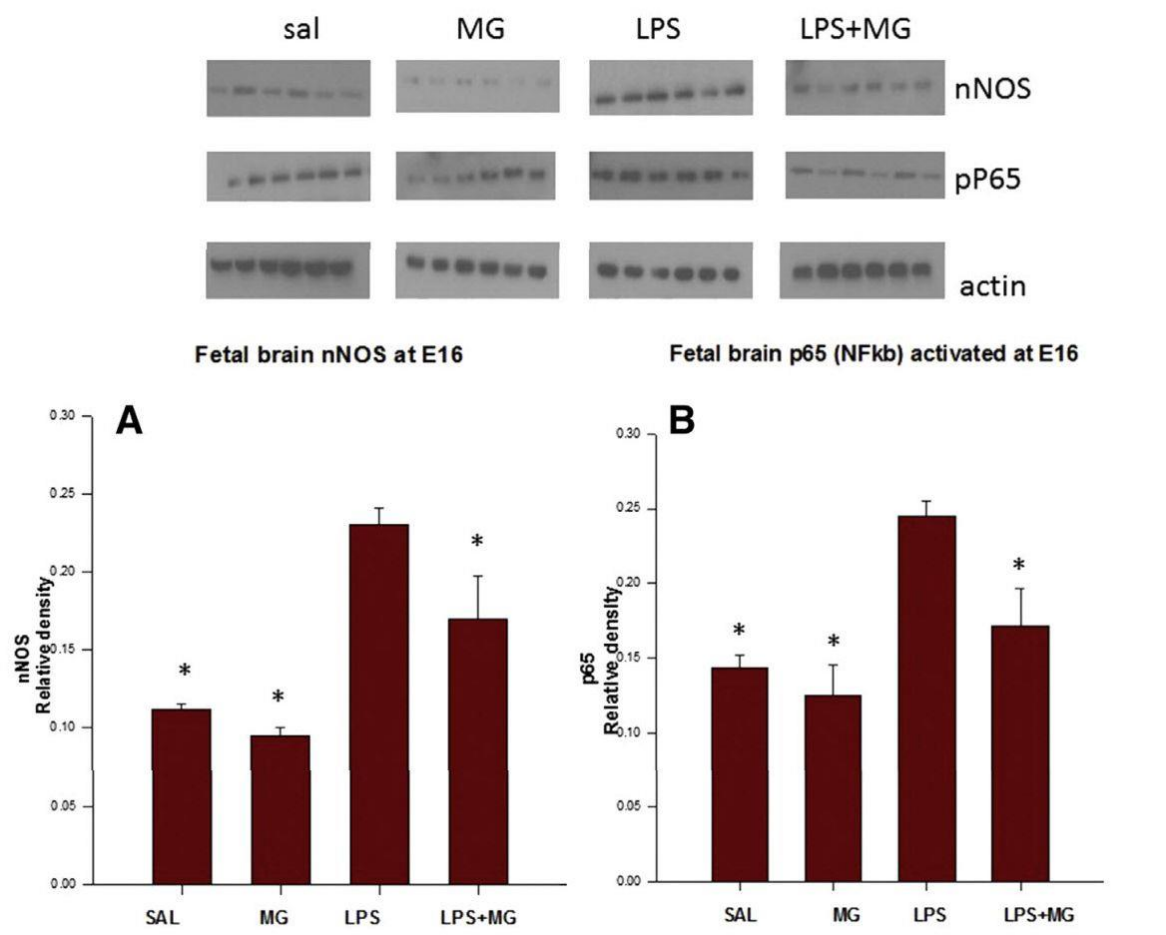
MgSO_4_ Decreases Levels of Fetal Brain nNOS and NF-κB Following Maternal Inflammation. **(A)** Neuronal nitric oxide synthase (nNOS). **(B)** Nuclear factor kappa-light-chain-enhancer of activated B cell (NF-κB) activation at embryonic day 16. Comparison between four treatment groups: normal saline (SAL); MG (magnesium sulfate); LPS (lipopolysaccharide); LPS-MG (4 hours following injection). *P<0.05 compared to the LPS group. Reprinted from Beloosesky et al.,^15^ Copyright (2016), with permission from Elsevier.

Recently we reported^15^ that MG treatment of LPS dams significantly decreased fetal brain phospho-nNOS, NF-κB, and CCL2 protein levels that presented subsequent to maternal administration of LPS. This study reports, for the first time, that acute maternal inflammation can induce an inflammatory response in the fetal brain through direct activation of fetal brain NF-κB and phospho-nNOS, and that maternal MG attenuates this response through N-methyl-D-aspartate receptor (NMDA-R) (Figure 4).

#### Fetal Brain Imaging Following Magnesium Sulfate Administration

**Figure 5:**
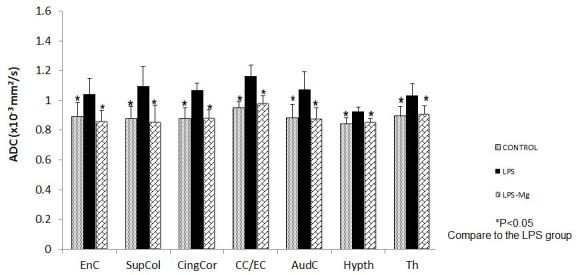
MgSO_4_ Decreases Fetal Brain Injury Following Maternal Inflammation. Neonatal brain MRI analysis at one month after delivery. Averaged ADC values at significant brain regions (see Table 1), in the LPS group, the LPS/MG group, and the Saline (control) group. *P<0.05 compared to the LPS group. AudC, auditory capsule; EnC, internal capsule; CC/EC, corpus callosum and external capsule; CingCor, cingulate cortex; Hypth, hypothalamus; LPS, lipopolysaccharide; MRI, magnetic resonance imaging; MG, magnesium sulfate; SAL, saline; SupCol, superior colliculus; Th, thalamus.

Maternal LPS at E18 induces brain injury in offspring at 25 days of age, evident by MRI.^18^ The injury was demonstrated in both gray and white matter areas. Magnesium sulfate treatment administered for 2 hours prior to and following i.p. LPS prevented offspring brain injury as demonstrated by MRI (Figure 5; Table 1).

**Table 1:** Affected Fetal Brain Region Function. Division of the affected brain region function (both white and gray matter) following the LPS-induced maternal inflammation. Comparison between the LPS and the Saline groups. Reprinted from Beloosesky et al.,^17^ Copyright (2013), with permission from Elsevier.

Motor Function	Learning and Memory System	Sensory Function	Emotional System	General
Ic (internal capsule)	CPu	S1 (the somatosensory system)	Cingo	CC
CPu (dorsal striatum)	MS (medial septal nucleus)	PirC	MS (medial septal nucleus)	Thalamic nucleus
M1 (the primary motor cortex)	Ect (entorhinal cortex)		IL/DTT (infralimbic cortex)	
EC	MoDG (molecular dentate gyrus		BLP (basolateral amygdaloid nucleus)	
	Sm (Stria medullaris of the thalamus)		Sm (stria medullaris of the thalamus)	

CC, corpus callosum; CPu, caudate putamen; EC, external capsule; LPS, lipopolysaccharide; NAC, N-acetyl-cysteine.

#### Learning and Memory Abilities

**Figure 6:**
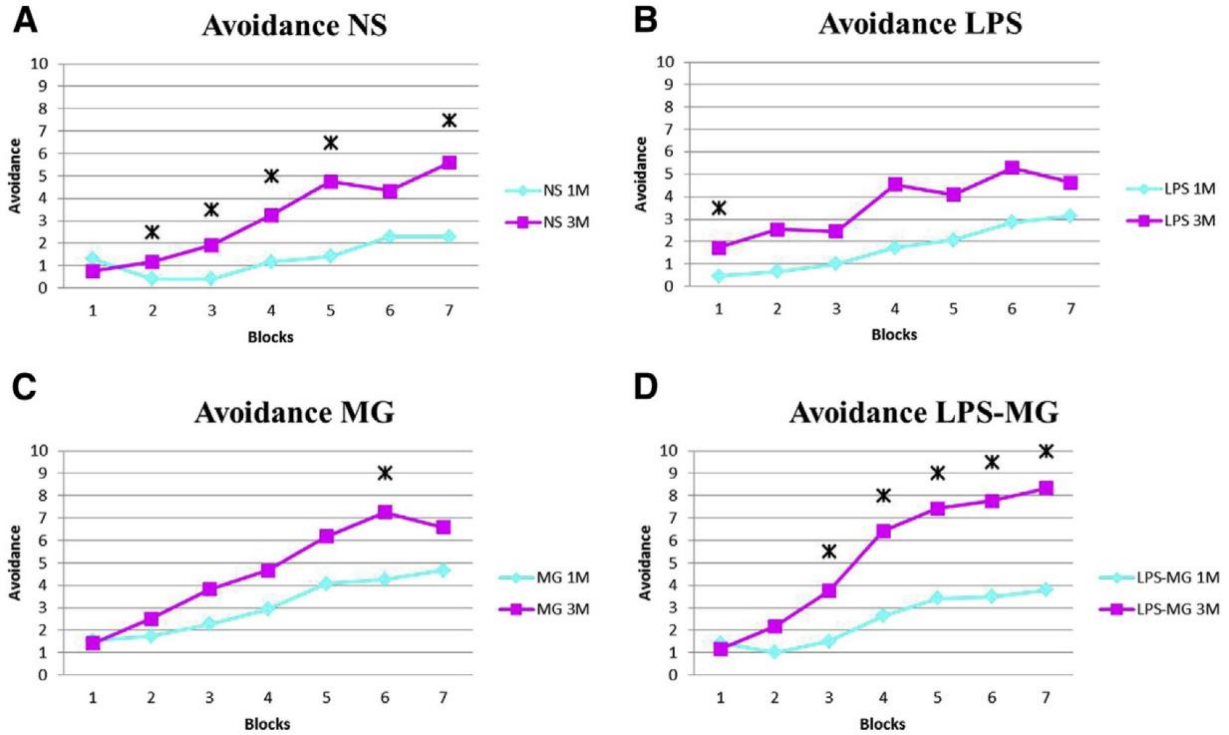
MgSO_4_ Improves Neonate Learning and Memory Abilities Following Maternal Inflammation. **(A)** Demonstration of significant improvement in learning abilities of the NS group, between the first and the third months in almost every block. **(B)** The LPS group exhibited no significant improvement with time. (**C)** The MG group displayed significantly better results than the NS group at 1 month of age; however, little improvement was seen at 3 months. (**D)** Magnesium treatment administered to LPS mothers significantly improved memory and learning abilities at both 1 and 3 months. LPS, lipopolysaccharide; MG, magnesium sulfate; NS, normal saline; 1M, 1 month of age; 3M, 3 months of age. Reprinted from Lamhot et al.,^23^ Copyright (2015), with permission from Elsevier. Asterisks (*): Significance (*P*<0.05) in the learning abilities, examined by the avoidance test in each block between 1 and 3 months. **Blocks (X Axis):** Each test comprised 7 blocks of 10 stimulus cycles each. Tests were administered to each rat at 1 and 3 months. **Avoidance (Y Axis):** Number of positive avoidance responses in each block.

As described before,^23^ we used the two-way shuttle avoidance test to investigate neurobehavioral outcomes associated with maternal inflammation and the impact of neuroprotection with MG. Our results demonstrated a clear decline in learning abilities of the offspring of LPS-treated dams and a return to normal values following treatment with MG. Interestingly, at age 3 months the abilities of the LPS-MG group exceeded those of all the other groups, including the control-saline and the MG-only group (Figure 6). The reason for this unexpected result is unclear.

Our results support the notion that MG protection is not limited to the white matter, but that it may protect the gray matter and cognitive functions of the offspring as well.

#### N-Acetyl Cysteine

N-acetyl cysteine (NAC) is an antioxidant that scavenges free oxygen radicals. In addition, NAC can act indirectly as a stimulant of synthesis of the anti-stressogenic agent glutathione.^52^,^53^ The effectiveness of NAC as a neuroprotective has been already well established. In a hypoxia-ischemia rat model, Wang et al.^54^ demonstrated that treatment with NAC significantly decreased brain injury in LPS-sensitized pups. As previously mentioned, since the inflammatory reaction might cause accumulation of both cytokines and oxygen free radicals, we thought to use NAC to reduce the inflammatory reaction and to narrow the risk of fetal brain injury.

#### Cytokines

**Figure 7:**
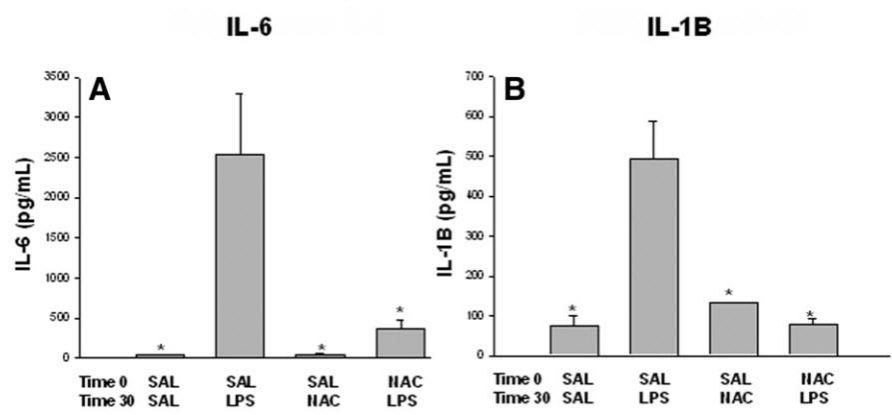
NAC Decreases Fetal Plasma IL-6 and IL-1β Levels Following Maternal Inflammation. Fetal plasma IL-6 and IL-1β levels after first injection. (**A)** Fetal plasma IL-6. (**B)** Fetal plasma IL-1β levels 6 hours after the first injection in the four treatment groups: SAL-SAL, SAL-LPS, SAL-NAC, and NAC-LPS. *P<0.05, significantly different from the SAL-LPS group. LPS, lipopolysaccharide; NAC, N-acetyl cysteine; SAL, saline. Reprinted from Beloosesky et al.,^21^ Copyright (2009), with permission from Elsevier.

Following LPS-induced maternal inflammation, NAC administration significantly reduced pro-inflammatory cytokine expression in the maternal circulation, amniotic fluid, fetal blood, and most importantly in the fetal brain (Figure 7).^14^,^16^,^17^,^20^

#### Fetal Brain Imaging Following NAC Administration

**Figure 8:**
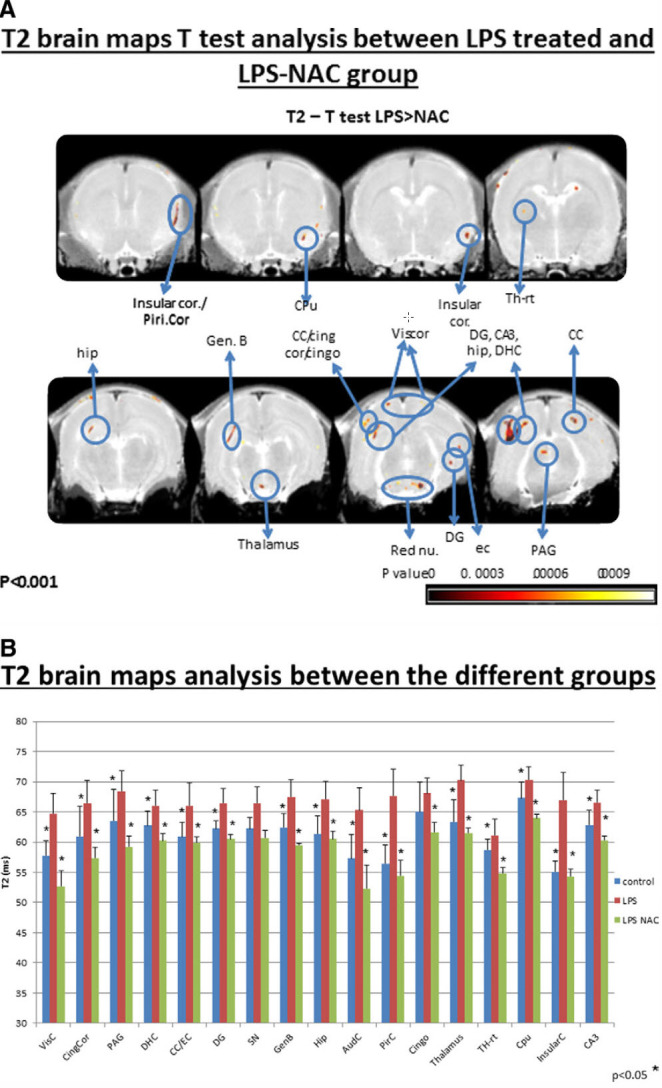
NAC Decreases Fetal Injury as Demonstrated by MRI Following Maternal Inflammation. T2 voxel-based *t* test analysis, comparing the LPS-treated and the LPS-NAC groups, both with neonatal brain MRI at day 25 after delivery. (**A)** Images of T2 MRI comparing the LPS group and the LPS-NAC group (higher T2 implies brain damage). The *colored areas* indicate significant differences in T2 between the LPS group and the LPS-NAC group. The significant regions are superimposed on T2 relaxation maps (*P*<0.001). (**B)** The graphs present averaged T2 values at significant brain regions in the LPS group, the LPS-NAC group, and the control group. The LPS group included SAL-LPS-SAL; the LPS-NAC group included NAC-LPS-NAC; the control included SAL-SAL-SAL. *P<0.05. LPS, lipopolysaccharide; MRI, magnetic resonance imaging; NAC, N-acetyl cysteine; SAL, saline. Reprinted from Beloosesky et al.,^17^ Copyright (2013), with permission from Elsevier.

Maternal NAC administration following the i.p. LPS-induced maternal inflammation prevented any visible damage to the offspring neonatal brains^17^ (Figure 8; Table 1).

Our findings suggest that NAC might attenuate the short- and long-term sequelae associated with a fetal brain inflammatory response in pregnant women affected by severe inflammation. Although these were encouraging results concerning the influence of NAC upon both fetal and maternal immune responses, further studies regarding this are still needed.

## CONCLUSION

Maternal inflammation during pregnancy is associated with an increased risk of neurodevelopmental disorders in the offspring. Activation and up-regulation of inflammatory cytokines and oxidative stress, both systemically and in the fetal brain, are thought to play a key role in altered brain development and may contribute to poor neurodevelopmental outcomes subsequent to maternal inflammation. Possible therapeutic intervention may include antioxidative and anti-inflammatory therapies aimed to reduce the severity and extent of the injury.

In order to develop realistic therapeutic options, future research is needed to clarify the mechanisms linking maternal inflammation, fetal brain development, and neurological and behavioral deficits.

## Abbreviations

ADC: apparent diffusion coefficient
DTI: diffusion tensor imaging
IL: interleukin
IL-1β: interleukin 1β
i.p.: intraperitoneal
LPS: lipopolysaccharide
MRI: magnetic resonance imaging
MG: magnesium sulfate
NAC: N-acetyl cysteine
NOS: nitric oxide synthase
NS: normal saline
NF-κB: nuclear factor kappa-light-chain-enhancer of activated B cells
NMDA-R: N-methyl-D-aspartate receptor
TNF-α: tumor necrosis factor-alpha
